# Long-term outcomes of anatomic vs. non-anatomic resection in intrahepatic cholangiocarcinoma with hepatolithiasis: A multicenter retrospective study

**DOI:** 10.3389/fmed.2023.1130692

**Published:** 2023-03-20

**Authors:** Jun-Yi Wu, Wen-Tao Huang, Wen-bin He, Gao-Fan Dai, Jia-Hui Lv, Fu-Nan Qiu

**Affiliations:** ^1^Shengli Clinical Medical College, Fujian Medical University, Fuzhou, China; ^2^Department of Hepatobiliary Pancreatic Surgery, Fujian Provincial Hospital, Fuzhou, China; ^3^Department of Surgical Intensive Care Unit, First Affiliated Hospital of Fujian Medical University, Fuzhou, China; ^4^Department of Hepatobiliary Surgery, Mengchao Hepatobiliary Hospital of Fujian Medical University, Fuzhou, China

**Keywords:** intrahepatic cholangiocarcinoma with hepatolithiasis, anatomic resections, overall survival, recurrence-free survival, lymph node metastases (LNM)

## Abstract

**Background:**

The benefits of anatomic resection (AR) vs. non-anatomic resection (NAR) in patients with primary intrahepatic cholangiocarcinoma (ICC) with hepatolithiasis (HICC) are unclear. This study aimed to compare the long-term outcomes of AR vs. NAR in patients with HICC.

**Methods:**

A total of 147 consecutive patients with HICC who underwent R0 hepatectomy were included. Overall survival (OS) and recurrence-free survival (RFS) following AR vs. NARs were compared using a 1:1 propensity score matching (PSM) analysis. A subgroup analysis was also conducted according to whether there are lymph node metastases (LNM).

**Results:**

In a multivariate analysis, CA 19-9 (>39 U/L), microvascular invasion, LNM, and NAR were independent risk factors for poor RFS and OS rates, whereas multiple tumors were independent risk factors for OS. AR had better 1-, 3-, and 5-year RFS and OS rates than NAR (OS: 78.7, 58.9, and 28.5%, respectively, vs. 61.2, 25.4, and 8.8%, respectively; RFS: 59.5, 36.5, and 20.5%, respectively, vs. 38.2, 12.1, and 6.9%, respectively). After PSM, 100 patients were enrolled. The NAR group also had significantly poorer OS and RFS (OS: 0.016; RFS: *p* = 0.010) than the AR group. The subgroup analysis demonstrated that in HICC without LNM, OS and RFS were significantly poorer in the NAR group than the AR group, while no significant differences were observed in HICC with LNM before or after PSM.

**Conclusion:**

Anatomic resection was associated with better long-term survival outcomes than NAR in patients with HICC, except for patients with LNM.

## Background

Intrahepatic cholangiocarcinoma (ICC) is the second most common primary hepatic malignancy (1, 2). The incidence of ICC has been reported to be increasing worldwide over the past decades (3). Hepatolithiasis is one of the multifactorial etiologies of ICC, which have a high prevalence in Asian countries (4). Several studies have indicated hepatolithiasis as an independent risk factor for patients with ICC, and the total incidence of ICC caused by hepatolithiasis is ~ 5–13% in Asian populations (5–7).

Liver resection is the first-line therapeutic option for patients with ICC, including those with ICC with hepatolithiasis (HICC), to achieve a possible long-term survival (8). Although many studies have focused on therapy methods for patients with ICC, the prognosis of these patients is dismal owing to high incidences of post-operative recurrence and metastasis (9, 10). Several studies have indicated that the 5-year overall survival (OS) of patients with ICC after curative resection was only 20–35% (9, 10). More importantly, patients with HICC had worse outcomes than those without hepatolithiasis (6, 11).

Anatomic resection (AR) has been recommended to be superior to liver resection in reducing the risk of post-operative intrahepatic recurrence in patients with hepatocellular carcinoma (HCC) (12, 13). However, the number of studies investigating post-hepatectomy OS between AR and non-anatomic resection (NAR) for ICC is limited (14, 15), and the conclusions are still controversial. To the best of our knowledge, no studies have investigated the long-term outcomes of AR and NAR for HICC. In this study, we aimed to compare the clinical outcomes of patients with HICC who underwent AR and NAR using the propensity score matching (PSM) analysis.

## Methods

### Patients

We retrospectively reviewed the data of patients with HICC who underwent R0 resection between October 2012 and December 2021 at the following three high-volume institutions: Fujian Provincial Hospital (Fuzhou, China), Mengchao Hepatobiliary Hospital of Fujian Medical University (Fuzhou, China), and the First Affiliated Hospital of Fujian Medical University (Fuzhou, China). The diagnosis of HICC was confirmed by two experienced pathologists who were dependent on the post-operative histopathological examination at each participating hospital. R0 resection was defined as complete tumor removal with a free microscopic margin. Data, including standard demographics, perioperative clinicopathological, and post-operative outcomes, were retrospectively collected. This study was approved by the Institutional Ethics Committee of Fujian Provincial Hospital. The ethical license number was K2022-07-011. All the participants provided written informed consent for the use of their data.

The inclusion criteria were as follows: (1) patients with HICC who underwent R0 resection, (2) with primary ICC lesions without contiguous organ invasion or extrahepatic metastasis, and (3) age of 18–75 years with good operative tolerance. The exclusion criteria were as follows: (1) combined with other serious malignant diseases (*n* = 3), (2) Child–Pugh class C liver function (*n* =1), (3) combined with macrovascular invasion (*n* = 16), (4) receiving pre-operative anticancer treatment (*n* = 4), (5) combined HCC and ICC (*n* = 28), (6) patients who died within 90 days of surgery (*n* = 3), (7) patients who died of other disease-related causes (*n* = 2), (9) non-R0 resection (*n* = 14), and (10) incomplete data (*n* = 8).

### Liver resection

Patients with obstructive jaundice (total bilirubin (TBil) level >200 μmol/L) or acute cholangitis were treated with percutaneous transhepatic biliary drainage that was placed in their contralateral intrahepatic bile duct to reduce the TBil level pre-operatively. The TBil criteria for surgery after PTCD was TBil level <50 μmol/L or cure for acute cholangitis. It was generally not more than 2 weeks. AR was classified as a liver resection based on the systematic removal of the Couinaud segment(s), which include the tumor together with the tumor-bearing portal vein and hepatic territory, and NAR was classified as all other resections that were not in accordance with the anatomical distribution of the portal vein branches. Regional lymphadenectomy was performed if lymph node metastasis was suspected or diagnosed either pre-operatively or intraoperatively. A choledochoscope was routinely used for exploration in all cases.

### Follow-up

Follow-up occurred once every 3 months for the first 2 years after the initial surgery and every 6 months thereafter. At each visit, tests for liver function (TBil, serum albumin, alanine aminotransferase, and aspartate aminotransferase), serum alpha-fetoprotein level (AFP), carbohydrate antigen 19-9 (CA 19-9), and carcinoembryonic antigen (CEA), as well as imaging examinations (contrast-enhanced computed tomography or magnetic resonance imaging) were performed. When recurrence was diagnosed, the treatment was decided based on the pattern of recurrence, liver functional reserve, and general condition of the patient.

The OS rate was calculated from the date of the first liver resection to the date of the patient's death or last follow-up. The recurrence-free survival (RFS) rate was the interval between the date of surgery and the date of diagnosis of the first recurrence or last follow-up.

### Statistical analyses

Data were analyzed using the SPSS software (version 17.0; SPSS, Inc., Chicago, IL, USA). Categorical variables were compared using the chi-square test or Fisher's exact test. Continuous variables were compared using the *t*-test or Mann–Whitney *U*-test. Univariate and multivariate comparisons of survival distributions were performed using Cox proportional hazard models, and factors with a *p* < 0.05 in the univariate analysis were then incorporated into the multivariate analysis. The OS and RFS rates between AR and NAR were calculated using the Kaplan–Meier method, and the significance of differences between the two groups was compared using the log-rank test. All *p*-values were two-sided and considered significant at a *p*-value of < 0.05.

A PSM analysis was performed to eliminate selection bias. The variables used in the PSM analysis included the following: tumor size, sex, age, hepatitis B surface antigen status (HBsAg), liver cirrhosis, Child–Pugh class, CEA, CA 19-9, tumor size, and tumor number. The PSM was performed *via* 1:1 matching with a caliper width of 0.02 of the standard deviation.

## Results

### Patient clinicopathological characteristics

Altogether, 147 patients with HICC who underwent R0 hepatectomy without macrovascular invasion, direct invasion to contiguous organs, or extrahepatic metastasis between October 2012 and December 2021 in the three institutions were included in our study. Of these patients, 80 (54.42%) and 67 (45.58%) patients underwent AR and NAR, respectively. The clinicopathological baseline characteristics of the patients with HICC are presented in Table 1. Of the 147 patients, 55 (37.41%) patients had LNM, 99 (67.35%) had tumors >5 cm in diameter, 32 (21.77%) had multiple tumors, and 49 (33.33%) presented with liver cirrhosis. Before PSM, the two groups showed a significant difference in liver cirrhosis. After PSM, there were no significant differences in cirrhosis.

**Table 1:** Patient demographics and tumor characteristics.

**Variables**	**Before PSM (*****n*** = **147)**			**After PSM (*****n*** = **100)**		
	**NAR (*****n*** = **67)**	**AR (*****n*** = **80)**	***P*** **-value**	**NAR (*****n*** = **50)**	**AR (*****n*** = **50)**	***P*** **-value**
Sex			0.278			0.689
Male	30	43		24	26	
Female	37	37		26	24	
Age (years)			0.827			0.373
≤65	48	56		34	38	
>65	19	24		16	12	
HBsAg			0.468			0.517
Yes	23	23		17	14	
No	44	57		33	36	
Anti-HCV			0.510			1.000
Yes	3	2		2	2	
No	64	78		48	48	
Liver cirrhosis			**0.047**			0.826
Yes	28	21		15	14	
No	39	59		35	36	
Tbil			0.324			0.603
≤23 μmol/L	57	63		42	40	
>23 μmol/L	10	17		8	10	
ALB			0.172			0.542
≤40 g/L	30	27		19	22	
>40 g/L	37	53		31	28	
ALT			0.224			0.668
≤40 U/L	49	51		35	33	
>40 U/L	18	29		15	17	
AST			0.797			0.260
≤40 U/L	49	60		39	34	
>40 U/L	18	29		11	16	
ALP			0.429			1.000
≤125 U/L	35	47		28	28	
>125 U/L	32	33		22	22	
GGT			0.787			0.840
≤60 U/L	27	34		21	22	
>60 U/L	49	46		29	28	
AFP			0.535			0.695
≤20 ng/mL	62	76		46	47	
>20 ng/mL	5	4		4	3	
CA19-9			0.329			0.529
≤39 U/L	24	35		19	16	
>39 U/L	43	45		31	34	
CEA			0.948			1.000
≤10 μg/L	55	66		40	40	
>10 μg/L	12	14		10	10	
Tumor number			0.539			0.617
Single	50	65		41	39	
Multiple	17	15		9	11	
Tumor diameter			0.244			0.683
≤5	23	35		21	19	
>5	44	45		29	31	
MVI			0.646			1.000
Yes	19	20		16	16	
No	48	60		34	34	
Nodal metastasis			0.508			0.545
Yes	27	28		20	23	
No	40	52		30	27	
Macroscopic type			0.345			0.275
MF	52	67		40	44	
Non-MF	15	13		10	6	
Tumor differentiation			0.771			0.817
Well/moderate	50	58		37	38	
Poor	17	22		13	12	
Operation time, min	206.5 ± 93.0	234.7 ± 82.4	0.053	208.9 ± 91.6	227.1 ± 83.3	0.300
Blood loss, mL median (range)	300 (50–2,450)	300 (50–2,500)	0.505	200 (50–2,450)	300 (100–2,500)	0.763
Post-operative hospital stays, days	13.7 ± 7.7	13.9 ± 7.2	0.913	13.3 ± 7.7	13.1 ± 5.9	0.840
Adjuvant chemoradiotherapy			0.580			0.305
Yes	23	31		17	22	
No	44	49		33	28	
Grade of complications			0.943			1.000
Non	35	44		23	23	
I–II	26	29		22	22	
III–IV	6	7		5	5	
Long-term outcome of stone recurrence			**0.039**			0.059
Yes	9	3		7	1	
No	58	77		43	49	

The bold values indicate *P* < 0.05, which had a significant difference between the two group.

In terms of post-operative characteristics, although the AR group had a slightly longer operative time than the NAR group, there were no significant differences in the operative time (Table 1). Post-operative hospital stay and operative blood loss were also not significantly different between both groups. Meanwhile, the incidence of grades I–II and III–IV surgical complications in the AR and NAR groups was similar (Table 1). More importantly, the AR could significantly reduce the rate of stone recurrence (*P* = 0.039). The long-term outcomes of stone recurrence after PSM (*P* = 0.059) did not significantly differ between the two groups, and this may be because of the small number of cases.

### Independent predictors of RFS and OS

Univariate analysis revealed that CA 19-9 (>39 U/L), tumor number (multiple), microvascular invasion (MVI; positive), LNM (positive), and AR (yes) were independent risk factors for OS and RFS rates. Maximum tumor size (>5.0 cm) was independently associated with RFS (Supplementary Table 1). Multivariate analysis revealed that CA 19-9 (>39 U/L), MVI (positive), LNM (positive), and AR (positive) were independent risk factors for OS and RFS rates, whereas tumor number (multiple) was an independent risk factor for OS (Table 2).

**Table 2:** Multivariate analysis of factors related to the RFS and OS before PSM.

**Variables**	**RFS**			**OS**		
	**HR**	**(95%CI)**	***P*** **-value**	**HR**	**(95%CI)**	***P*** **-value**
ALP (>125 U/L)	0.899	0.595–1.357	0.612			
CA19-9 (>39 U/L)	1.858	1.210–2.854	**0.005**	1.996	1.249–3.189	**0.004**
Tumor number (multiple)	1.611	0.987–2.628	0.056	2.654	1.591–4.427	**<0.001**
Tumor size (>5 cm)	1.254	0.817–1.925	0.301	1.076	0.689–1.681	0.748
MVI (positive)	2.282	1.484–3.510	**<0.001**	1.874	1.187–2.959	**0.007**
Nodal metastasis (positive)	1.849	1.194–2.863	**0.006**	2.432	1.536–3.852	**<0.001**
AR (yes)	2.008	1.370–2.943	**<0.001**	2.237	1.477–3.390	**<0.001**

The bold values indicate *P* < 0.05, which had a significant difference between the two group.

### Long-term outcomes

**Figure 1:**
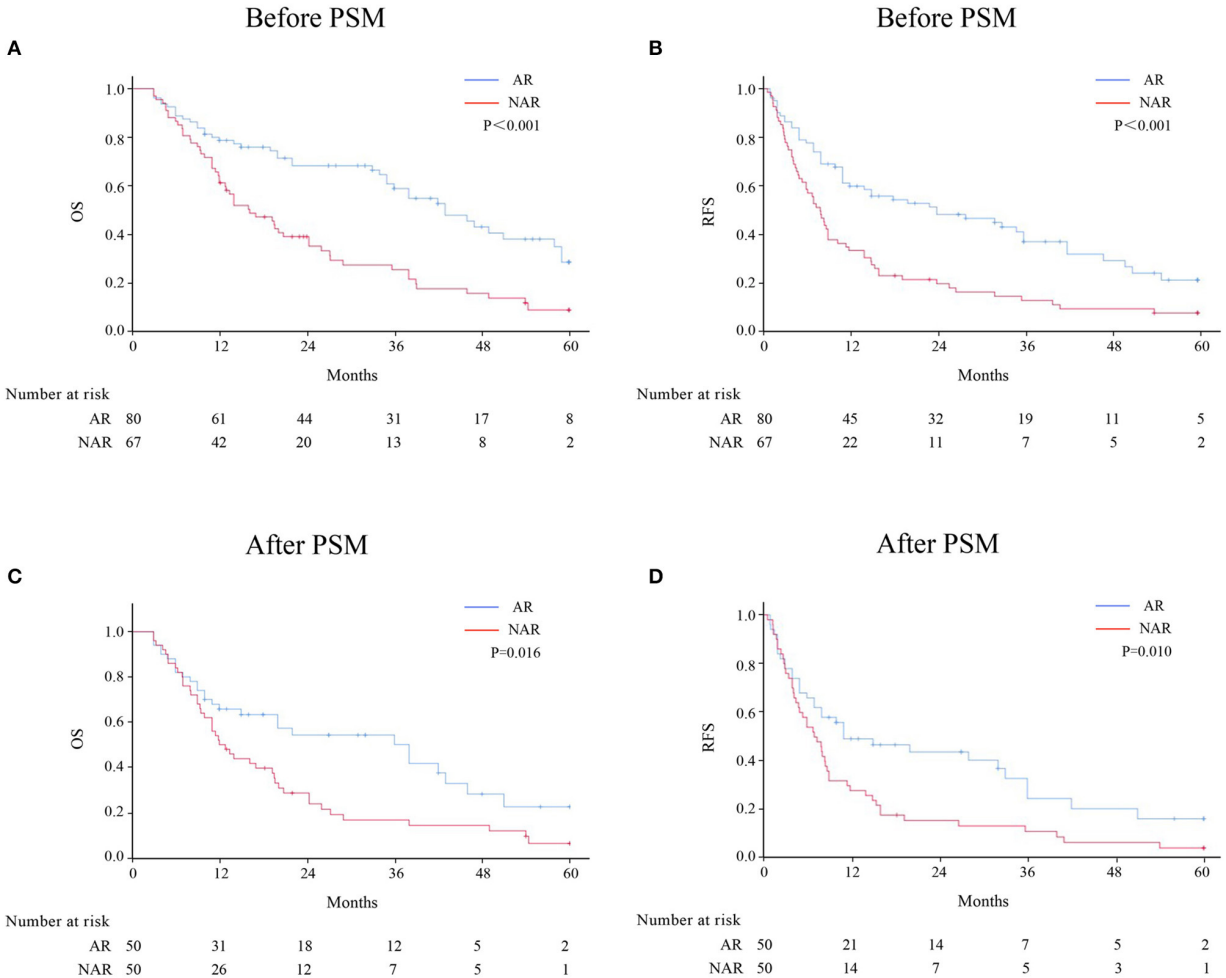
OS and RFS rates after AR vs. NAR for patients with HICC before **(A, B)** and after PSM **(C, D)**.

Before PSM, the 1-, 3-, and 5-year OS rates for patients with HICC were 78.7, 58.9, and 28.5%, respectively, in the AR group, and 61.2, 25.4, and 8.8%, respectively, in the NAR group (*p* < 0.001) (Figure 1A). The 1-, 3-, and 5-year RFS rates were 59.5, 36.5, and 20.5%, respectively, in the AR group, and 38.2, 12.1, and 6.9%, respectively, in the NAR group (*p* < 0.001) (Figure 1B). After PSM, AR was associated with better 1-, 3-, and 5-year RFS (Figure 1C; 1 year, 49.2 vs. 28.0%; 3 years, 24.7 vs. 11.2%; and 5 years, 16.5 vs. 4.5%; *p* = 0.010) and OS rates (Figure 1D; OS, 65.8 vs. 52.0%; 3 years, 50.1 vs. 16.5%; and 5 years, 22.5 vs. 6.3%; *p* = 0.016) than NAR.

### Subgroup analysis of survival according to lymph node metastases

**Figure 2:**
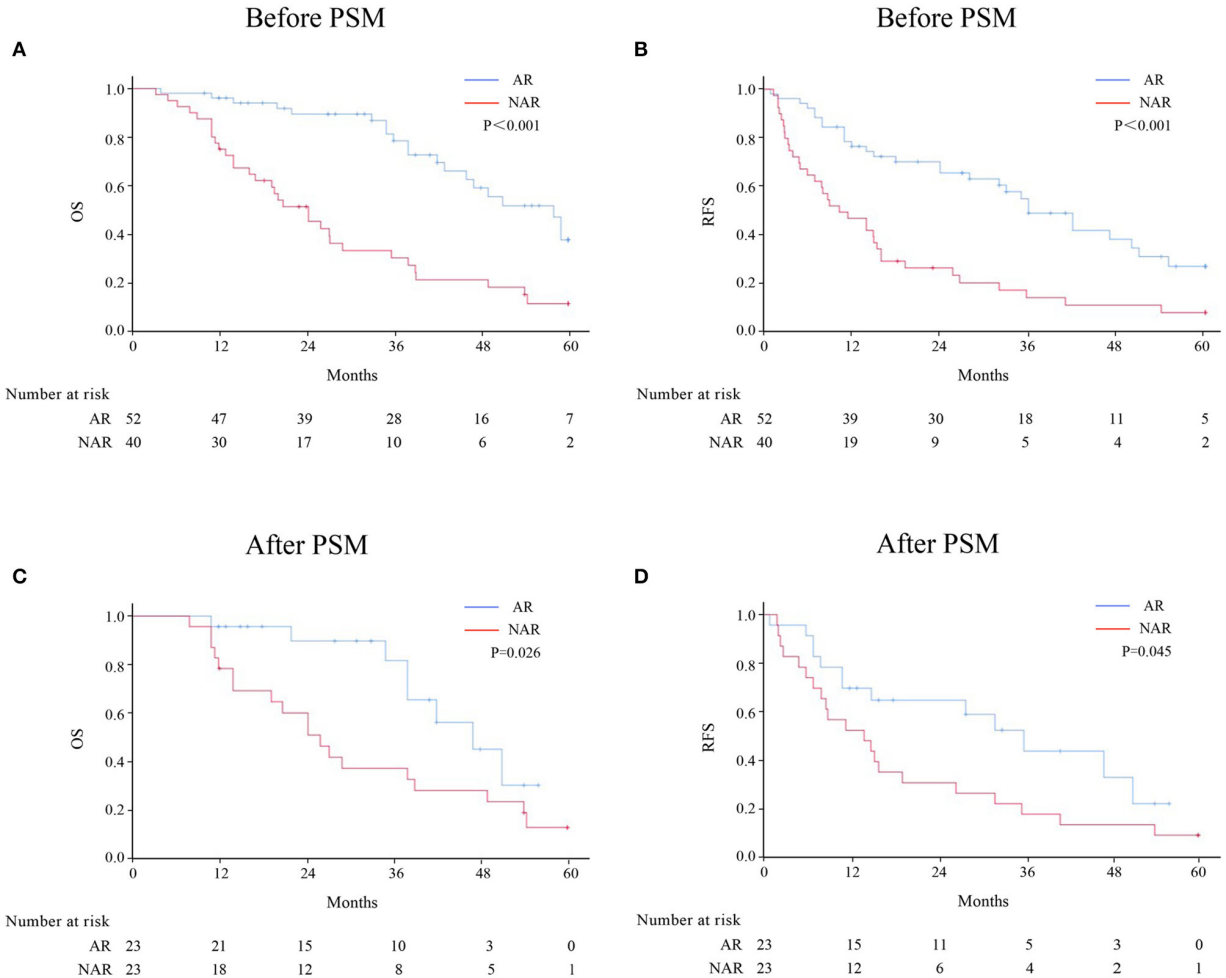
OS and RFS rates after AR vs. NAR for HICC patients without LNM before **(A, B)** and after PSM **(C, D)**.

**Figure 3:**
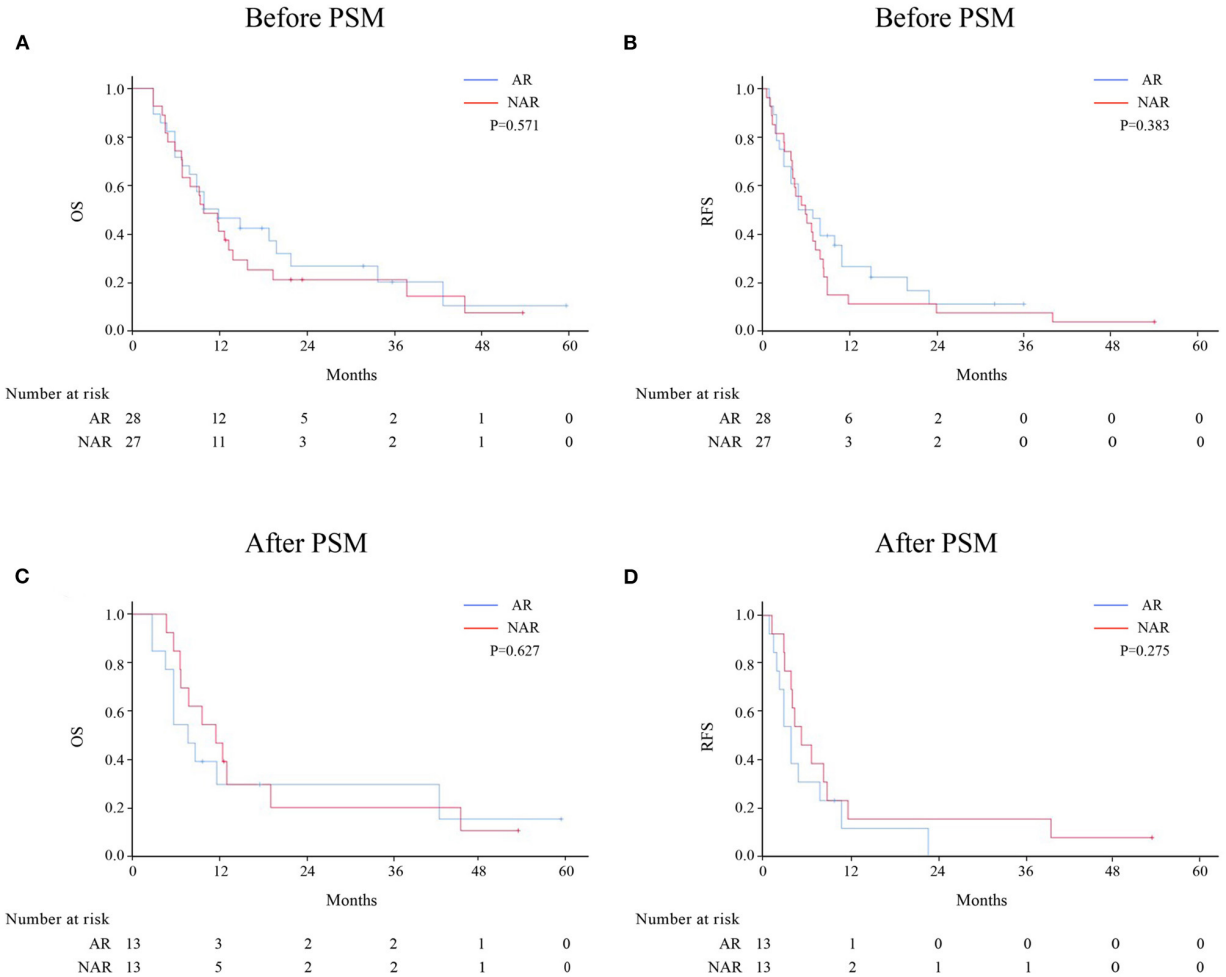
OS and RFS rates after AR vs. NAR for HICC patients with LNM before **(A, B)** and after PSM **(C, D)**.

Patients with HICC were sub-categorized according to LNM (Figure 2). Among 92 patients without LNM, the AR group demonstrated better OS and RFS rates than the NAR group before and after PSM. However, no significant difference was observed between both groups among HICC patients with LNM (Figure 3; before PSM, OS: *p* = 0.571, RFS: *p* = 0.383; after PSM, OS: *p* = 0.627, RFS: *p* = 0.275, respectively).

## Discussion

To date, a series of studies have indicated that patients with ICC who underwent partial hepatectomy still had a low 5-year OS rate (2, 3). As for those with HICC, their prognosis was poorer than patients with ICC without hepatolithiasis (16, 17). Hepatolithiasis frequently results in the development of atypical epithelium, oncogene activation, and inflammation, leading to the high occurrence of periductal invasion and LNM, which leads to a poor prognosis (18, 19). However, the early symptoms of HICC are not typical and can be easily concealed by intrahepatic bile duct stones and cholangitis. The sensitivity and specificity of laboratory tests and imaging studies for HICC are relatively low, which leads to a delay in diagnosing HICC and the advanced tumor stage (19). In our data, 42 (28.57%) of the patients with HICC were diagnosed by pathological testing after partial hepatectomy. Therefore, surgeons should consider the possibility of co-existing ICC when performing surgery on patients with hepatolithiasis.

Although patients with HICC had a very poor prognosis, curative resection remains the best curative treatment for HICC (20). Previous studies have demonstrated that AR was associated with better survival outcomes than NAR for HCC lesions, with AR considered theoretically effective in reducing intrahepatic recurrence (21, 22). Although both HCC and ICC arise in the hepatic parenchyma, the impact of AR on the prognosis for ICC remains unclear. Moreover, studies on the benefit of AR for ICC are limited, and their conclusions are inconsistent (14, 15). Li et al. concluded that NAR was not inferior to AR in improving the survival outcomes of patients with ICC. In contrast, Si et al. have reported that AR was associated with a better prognosis than NAR in patients with ICC with stage IB or II without vascular invasion. However, no prospective studies have compared the clinical outcomes of patients with HICC who underwent AR and NAR, and the surgical method of operation for patients with HICC has not been extensively researched. Previous studies have reported that AR was effective for treating hepatolithiasis and was associated with a low rate of recurrence (23, 24). Thus, patients with HICC may benefit from AR.

In the present study, AR conferred better OS and RFS outcomes than NAR in patients with HICC who underwent R0 hepatectomy without macrovascular invasion, direct invasion to contiguous organs, or extrahepatic metastasis. In addition, AR could significantly reduce the rate of stone recurrence before PSM. Multivariate analyses revealed AR as an independent favorable prognostic factor for OS and RFS. Subgroup analyses further demonstrated that HICC patients without LNM would receive more benefits from AR than that from NAR. Meanwhile, no significant difference between AR and NAR was observed in HICC patients with LNM.

Several studies have indicated that NAR is generally suitable for patients with poor liver function or liver cirrhosis (25, 26). Poor liver function and liver cirrhosis are limiting factors for extensive liver resection in patients with ICC. The use of AR in patients with poor liver function or liver cirrhosis should still be assessed carefully to avoid liver failure post-operatively. In our study, the AR group comprised a few patients with liver cirrhosis. The different proportions of liver cirrhosis may be attributable to inconsistent results. Therefore, we used PSM to minimize the selection bias between the two groups. Moreover, our study demonstrated that the intraoperative bleeding, operative time, post-operative hospital stays, and grade of complications did not differ significantly between the AR and NAR groups. This may be due to the technological advances in hepatectomy and the selection of the most appropriate treatment for patients with ICC.

The relationship between LNM and the prognosis of ICC has been indicated in previous studies (27, 28). Nodal metastasis is generally believed to greatly influence the prognosis of patients with ICC compared with other risk factors (27). ICC patients with LNM had a significantly worse prognosis than those without LNM (27, 28). In the present study, the data demonstrated that the LNM of HICC, rather than the resection type, influenced long-term outcomes.

This study has several limitations. First, this was a retrospective study. Although we used PSM, biases in patient selection may still exist. Second, some patients who had normal lymph nodes that were not identified in the pre-operative imaging or surgical exploration did not undergo lymphadenectomy. Nevertheless, all patients with ICC were recommended to undergo lymphadenectomy. Third, the sample size was small. Thus, more randomized controlled trials with a large sample size are necessary to confirm our results.

## Conclusion

In conclusion, our study indicated that AR was associated with better survival outcomes than NAR in HICC patients without LNM.

## Data availability statement

The original contributions presented in the study are included in the article/Supplementary material, further inquiries can be directed to the corresponding author.

## Ethics statement

The studies involving human participants were reviewed and approved by the Ethics Committee of Fujian Provincial Hospital, Mengchao Hepatobilary Hospital of Fujian Medical University, and The First Affiliated Hospital of Fujian Medical University. All study participants gave their written informed consent. The patients/participants provided their written informed consent to participate in this study.

## Author contributions

F-NQ and J-YW: conceived and designed the research and drafted the manuscript. J-YW, W-TH, and W-bH: data acquisition. J-YW, W-TH, J-HL, and G-FD: data analysis. All authors read and approved the final manuscript.
