# Supplementary material for: Long-term outcomes of anatomic vs. non-anatomic resection in intrahepatic cholangiocarcinoma with hepatolithiasis: A multicenter retrospective study
Source: Front Med (Lausanne). 2023 Mar 20;10:1130692. doi: 10.3389/fmed.2023.1130692 (PMC10067634; doi:10.3389/fmed.2023.1130692)
Supplement: Supplementary file 1 [file Table_1.docx]

| Supplementary table 1 Univariate analysis of factors related to the RFS and OS before propensity score matching | | | | | | | |
| --- | --- | --- | --- | --- | --- | --- | --- |
| Variables | RFS | | |  | OS | | |
|  | HR | (95%CI) | P-value |  | HR | (95%CI) | P-value |
| Sex (male) | 0.948 | 0.655-1.372 | 0.777 |  | 1.118 | 0.749-1.669 | 0.584 |
| Age (>65 yr) | 1.142 | 0.761-1.715 | 0.686 |  | 1.041 | 0.666-1.626 | 0.860 |
| HBsAg (positive) | 0.948 | 0.634-1.419 | 0.763 |  | 0.917 | 0.590-1.426 | 0.701 |
| Anti-HCV (positive) | 0.661 | 0.210-2.083 | 0.479 |  | 0.975 | 0.308-3.088 | 0.966 |
| Liver cirrhosis (positive) | 1.302 | 0.880-1.925 | 0.243 |  | 1.156 | 0.752-1.776 | 0.508 |
| Tbil (> 23 μmol/L) | 0.727 | 0.438-1.206 | 0.217 |  | 0.961 | 0.574-1.609 | 0.880 |
| ALB (>40 g/L) | 0.889 | 0.608-1.298 | 0.542 |  | 0.884 | 0.587-1.331 | 0.554 |
| ALT (> 40 U/L) | 1.108 | 0.751-1.635 | 0.605 |  | 0.915 | 0.596-1.405 | 0.685 |
| AST (> 40 U/L) | 1.309 | 0.866-1.976 | 0.201 |  | 1.072 | 0.679-1.692 | 0.766 |
| ALP (> 125 U/L) | 1.438 | 0.993-2.082 | **0.054** |  | 1.310 | 0.877-1.957 | 0.187 |
| GGT (> 60 U/L) | 1.187 | 0.812-1.736 | 0.376 |  | 1.147 | 0.758-1.736 | 0.515 |
| AFP (> 20 ng/mL) | 0.921 | 0.428-1.983 | 0.834 |  | 1.025 | 0.448-2.343 | 0.954 |
| CA19-9 (> 39 U/L) | 2.300 | 1.544-3.425 | **<0.001** |  | 2.655 | 1.706-4.131 | **<0.001** |
| CEA (> 10 U/L) | 1.183 | 0.730-1.919 | 0.495 |  | 1.187 | 0.709-1.986 | 0.514 |
| Tumor number (multiple) | 1.928 | 1.190-3.124 | **0.008** |  | 2.806 | 1.708-4.607 | **<0.001** |
| Tumor diameter (> 5 cm) | 1.484 | 1.010-2.181 | **0.045** |  | 1.492 | 0.983-2.266 | **0.060** |
| MVI (positive) | 2.414 | 1.592-3.662 | **<0.001** |  | 2.026 | 1.302-3.153 | **0.002** |
| Nodal metastasis (positive) | 2.869 | 1.941-4.241 | **<0.001** |  | 3.415 | 2.247-5.189 | **<0.001** |
| Macroscopic type (MF) | 0.866 | 0.534-1.404 | 0.559 |  | 1.077 | 0.637-1.819 | 0.783 |
| Tumor differentiation (poor) | 0.876 | 0.570-1.346 | 0.546 |  | 0.819 | 0.512-1.310 | 0.405 |
| AR（Yes） | 2.095 | 1.442-3.045 | **<0.001** |  | 2.297 | 1.525-3.459 | **<0.001** |
